# Clinical presentation, risk factors and pathogens involved in bacteriuria of pregnant women attending antenatal clinic of 3 hospitals in a developing country: a cross sectional analytic study

**DOI:** 10.1186/s12884-019-2290-y

**Published:** 2019-04-29

**Authors:** Charlotte Tchente Nguefack, Cecile Okalla Ebongue, Chrystelle Nouwe Chokotheu, Cedric Ebong Ewougo, Théophile Nana Njamen, Emile Mboudou

**Affiliations:** 1 Depatment of Obstetrics and Gynecology, Faculty of Medicine and Pharmacological Sciences, University of Douala; Douala General Hospital, Po Box 303, Douala, Cameroon; 2 Douala General Hospital; Department of Biological Sciences, Faculty of Medicine and Pharmacological Sciences, University of Douala, Douala, Cameroon; 3 Faculty of Medicine and Pharmacological Sciences, University of Douala, Douala, Cameroon; 4 Master Clinical Biology, Faculty of Medicine and Pharmacological Sciences, University of Douala, Douala, Cameroon; 5 Douala General Hospital; Department of Obstetrics and Gynecology, Faculty of Health Sciences, University of Buea, Buéa, Cameroon; 6 Department of Obstetrics and Gynecology, Faculty of Medicine and Biomedical Science, University of Yaoundé Cameroon, Gyneco-Obstetric and Pediatric Hospital, Douala, Cameroon

**Keywords:** Bacteriuria, Pregnancy, Clinical presentation, Risk factors, Pathogens

## Abstract

**Background:**

Pregnancy increases the risk of recurrent bacteriuria and acute pyelonephritis which is associated with significant maternal and fetal risks. The prevalence of bacteriuria varies worldwide. Clinical diagnosis is challenging since it is usually mistaken for normal physiological changes during pregnancy. This study aims to determine the prevalence, clinical presentation, risk factors and microorganism responsible for bacteriuria in pregnant women of Douala city, Cameroun.

**Methods:**

A cross-sectional study was conducted in 3 hospitals of Douala from January to April 2015. We consecutively recruited all consented pregnant women aged 18 years and above attending antenatal clinics. Socio-demographic characteristics, medical and obstetrical past history, clinical signs and obstetric characteristics of the index pregnancy were collected. Thereafter, urine were collected aseptically and subjected to routine macroscopy, microscopy examination and culture. The culture was obtained by inoculation of 10 μl of urine on the appropriate medium. Identification of pathogens was done automatically using the VITEK2**™** (BioMérieux- France). Data were processed using the Statistical Package for the Social Sciences (SPSS) 18. Statistics were descriptive and analytic; Odds ratios were calculated. Associations between variables and bacteriuria were conducted using the Chi squared test and the fisher exact probability. Associations with *p*-values < 0.05 were considered statistically significant.

**Results:**

Overall, 354 pregnant women were enrolled with mean of age 28.18 ± 4.4. The prevalence of significant bacteriuria was 9.9% (35 out of 354). The prevalence of bacteriuria in women who were asymptomatic was 5.7%. Cystitis and pyelo-nephritis were observed in 3.6 and 0.6% respectively. The most commonly isolated organism was *Escherichia coli* (*E. coli)*: 48.6%. History of Urinary Tract Infection (UTI) (*p* = 0.035, OR = 2.183, CI = 1.055–4.518) was significantly associated with bacteriuria. High level of education was protective.

**Conclusions:**

Bacteriuria was frequent in pregnant women and significantly increased with the past history of UTI and low level of education. Asymptomatic bacteriuria was more common. *E coli* was the most frequent uropathogen. Education and proper treatment of UTI should be provided to reduce the burden of this pathology in order to prevent its severe complications.

## Background

Bacteriuria is the presence and the growth of microorganisms in the urinary tract. The range of clinical effect varies from asymptomatic bacteriuria (ABU) to urinary tract infection (UTI) (cystitis or acute pyelonephritis) [1]. UTI is a common health problem among women due to the anatomy of the urinary tract. Pregnancy increases the risk of recurrent bacteriuria and acute pyelonephritis due to the compression of ureters by gravid uterus causing stasis of urine flow. Hormonal and immunological changes in pregnancy are other contributing factors: high level of progesterone secretion which leads to stasis and decreases immunity. Physiological proteinuria and glycosuria promote microorganism growth in the urine of pregnant woman. [1–3].

Women with bacteriuria during pregnancy may be more likely to deliver pre-mature or low-birth-weight infants leading to increase neonatal mortality and morbidity [1, 3]. Without treatment, as many as 20 to 30% of pregnant women with ABU will develop a symptomatic urinary tract infection (cystitis and pyelonephritis) during pregnancy [1, 3, 4]. Acute pyelonephritis has been associated with anemia, pre-eclampsia, sepsis and chronic renal disease [5, 6].

The prevalence of bacteriuria in pregnancy varies worldwide. Asymptomatic bacteriuria occurs in 2 to 7% of pregnant women [1, 2]. This prevalence can be up to 30% in studies conducted in developing countries [7–13]. Acute cystitis occurs in approximately 1 to 2% of pregnant women, and the estimated incidence of acute pyelonephritis during pregnancy is 0.5 to 2% [2, 5].

One of the risk factors for bacteriuria is age. In healthy women, the prevalence for bacteriuria increases with age from about 1% in females aged 5 to 14 years to more than 20% in women at least 80 years of age [14]. Other risk factors for bacteriuria or UTI included the presence of genitourinary abnormalities (kidney, ureteral and bladder stones, tumors, urethral strictures, vesico-ureteric reflux) [2]. In pregnant women, risk factors for UTI included anemia, sexual activity, lower socioeconomic classes and past history of UTI [2, 15]. Age, multiparity and gestational age are not identified as risk factors for UTI during pregnancy in some recent studies [11, 15, 16].

To the best of our knowledge, there are few studies conducted on bacteriuria during pregnancy in Cameroon where urine culture is expensive. More over dipstick screening tests routinely used in pregnancy are focused mainly on the presence of glucose and protein in urine rather than indicators of bacteriuria. This means that, bacteriuria especially when asymptomatic is not considered as an essential part of antenatal care by many clinicians. Therefore, nothing is known regarding the epidemiology of bacteriuria in pregnant women in Douala Cameroon. This study therefore aimed to determine the prevalence of bacteriuria, associated risk factors and pathogens involved in pregnant women attending antenatal clinic of 3 Hospitals in Douala.

## Methods

A cross-sectional study was conducted at general hospital, Cité des palmiers and Deido districts hospitals from January to April 2015. These hospitals are located in Douala which is the economic capital of Cameroon.

We consecutively recruited all pregnant women aged 18 years and above attending antenatal clinics at the selected health facilities. They should not have been on antibiotics for at least 72 h and consented voluntary to participate. Socio-demographic characteristics, medical and obstetrical past history, clinical signs and obstetric characteristics of the index pregnancy were collected using a structured questionnaire. Gestational age of the index pregnancy was calculated using an early ultrasound scan or the first day of the last menstrual period.

### Urine collection and culture

After completing the interview, women were given disposable sterile containers by the interviewer, and counselled on how to collect midstream random voided urine while respecting asepsis. Approximately 20 ml of urine were collected. The urine samples were transported to the laboratory of Douala General Hospital for processing, within 1 hour of collection. We did not have a method to preserve the urine. If not sent within that period, it was discarded and the patient asked to collect again. The samples were subjected to routine macroscopy examination, culture and microscopy examination by lab technicians and main investigator supervised by Doctors in clinical biology. Macroscopy examination consisted of description of the urine sample with naked eyes. Each sample was used for microscopy and culture examination. Direct microscopy involved examining urine for the presence of pus cells, red blood cells, parasites, casts and epithelial cells. Microscopic analysis was also done after staining of the sediment. Gram negative bacteria stained pink, while gram positive bacteria stained violet.

The culture was obtained by inoculation of 10 μl of non centrifuged urine on the Cystine Lysine Electrolyte Deficient (CLED) and Eosine Methylen Blue (EMB) medium. The media were then incubated at 37 °C for 24 h. Bacterial growth was categorized and interpreted on the basis of colony forming units (CFU) as follows: ≥ 105 CFU/ml was considered positive. Identification of pathogens was done using automatic colorimetric method in the VITEK2**™** (BioMérieux- France).

### Data management and statistical analysis

Data obtained from this study were entered into the Census and Survey Processing System 5 (CSPRO 5) and processed using the SPSS 18. Quantitative variables were presented in to the central tendency data (average) and their dispersion (standard deviation, minimum, maximum). Categorical variables were presented in numbers and or percentages. Odds ratios (OR) were calculated using the maximum likelihood estimation technique. Associations between variables and bacteriuria were conducted using the Chi squared test at the 95% significant level. Fisher exact probability was determined in the case of dichotomous variables. Associations with *p*-values < 0.05 were considered statistically significant.

Variables tested for associated with bacteriuria were: age; marital status; education level; monthly income; the body mass index; number of sexual intercourse per week; gestational age; parity; Human Immunodeficiency Virus (HIV) status; history of urinary tract infection, abortion and premature delivery .

### Ethical issues

The institutional ethics committee of the research on health of the University of Douala gave ethical approval (reference number CEI-UD/139/02/2015/T) for the study and permission was obtained from Cité des Palmiers health district, Deido district Hospital and Douala general hospital.

## Results

Single urine samples were collected from a total of 354 pregnant women. Of the 354 pregnant women tested, 35 had significant bacteriuria giving a prevalence of 9.9%.The prevalence of bacteriuria in women who were asymptomatic was 5.7%. Cystitis and pyelo-nephritis were observed in 3.6 and 0.6% respectively.

The most commonly isolated pathogen was *E. coli (*48.6%), followed by *Klebsiella pneumoniae* (14.3%). Other isolates were *Enterobacter cloacae* (11.4%) and *Staphylococcus aureus* (8.6%) as seen in Table 1.

**Table 1:** Pathogens identified in pregnant women with bacteriuria

Germs	Number	Percentage
*Escherichia coli*	17	48.6
*Klebsiella pneumoniae*	5	14.3
*Enterobacter cloacae*	4	11.4
*Staphylococcus aureus*	3	8.6
*Staphylococcus xylosus*	2	5.7
*Serratia odorifera*	1	2.9
*Enterobacter aerogenes*	1	2.9
*Proteus mirabilis*	1	2.9
*Streptococcus sp*	1	2.9
Total	35	100

The characteristics of the 354 pregnant women are found in Tables 2 and 3. The mean age of the study participants was 28.18 ± 4.4 (range 18-42 years). They were mostly aged 23–32 years (67.2%), had attained secondary school (88.2%), were married (65.3%), didn’t have personal income (55.6%), were nulliparus or primiparus (65%) and in the third trimester of pregnancy (41.8%).

**Table 2:** Association between socio-demographic factors and bacteriuria in pregnancy

characteristics	bacteriuria			OR (IC à 95%)	p
	positive	negative	total		
Age					
18–22	5(11.1)	40(88. 9)	45(12.7)	1	
23–27	12(10.2)	106(89.8)	118(33.3)	0.906 (0.3–2.734)	0.86
28–32	13(10.8)	107(89.2)	120(33.9)	0.972 (0.326–2.901)	0.95
33–37	3(5.4)	53(94.6)	56(15.8)	0.453 (0.102–2.008)	0.29
38–42	2(13.3)	13(86.7)	15(4.2)	1.231 (0.213–7.12)	0.81
Marital status					
Single	17(13.8)	106(86.2)	123(65.3)		
Married	18(7.8)	213(92.2)	231(34.7)	0.537 (0.252–1.145)	0.11
Education					
illiterate	2(50)	2(50)	4(1.1)	1	
Primary	3(7.9)	35(92.1)	38(10.7)	0.088 (0.009–0.872)	**0.037**
Secondary	14(10.1)	124(89.9)	138(39)	0.113 (0.015–0.874)	**0.036**
University	16(9.2)	158(90.8)	174(49.2)	0.106 (0.014–0.81)	**0.03**
Income /Month					
No income	17(8.6)	180(91.4)	197(55.6)	1	
≤ 35,000	5(10.6)	42(89.4)	47(13.3)	1.237 (0.409–3.74)	0.70
> 35,000	13(11.8)	97(88.2)	110(31.1)	0.738 (0.255–2.135)	0.57

The significance of entries in boldface: significant *p* value

**Table 3:** Association between obstetrical factors and urinary tract infection

Characteristics	Bacteriuria Positive	Negative	Total	OR (CI: 95%)	P
Gestational age					
1st Trimester	5(6.9)	67(93.1)	72(20.3)	1	
2nd Trimester	16(11.9)	118(88.1)	134(37.9)	0.82 (0.43–1.57)	0.55
3rd Trimester	14(9.5)	143(90.5)	148(41.8)	0.75 (0.40–1.42)	0.37
Parity					
Parity 0	17(11.9)	126(88.1)	143(40.4)	1	
Parity 1	8(9.2)	79(90.8)	87(24.6)	0.797 (0.317–2.003)	0.62
Parity 2	5(7.6)	61(92.4)	66(18.6)	0.65 (0.22–1.919)	0.43
Parity 3	2(5.4)	35(94.6)	37(10.5)	0.486 (0.096–2.454)	0.38
Parity 4	2(13.3)	13(86.7)	15(4.2)	1.362 (0.238–7.783)	0.72
Parity 5	1(20)	4(80)	5(1.4)	2.207 (0.206–23.605)	0.51
Parity 6	0(0.0)	1(100)	1(0.3)	0.023 (0.001–129.25)	0.99

The assessment of associated factors to bacteriuria showed that history of UTI (*p* = 0.035, OR = 2.183, CI = 1.055–4.518) was significantly associated with bacteriuria. High level of education was protective: primary level (*p* = 0.037; OR = 0.088; IC = 0.009–0.872) secondary level (*p* = 0.036; OR = 0.113; IC = 0.015–0.874) and university level (*p* = 0.03; OR = 0.106; IC = 0.014–0.81). Other factors were not statistically significant predictor of bacteriuria in pregnancy. However, women who had 4 or 5 pregnancies (OR = 1.362; CI = 0.238–7.783 and OR 2.207; CI = 0.206–23.605) and those who have low salaries (OR = 1.237; 95%CI = 0.409–3.740) tended to be more likely to have bacteriuria when compared to those who did not meet these criteria (Tables 2,3,4).

**Table 4:** Association between medical and obstetrical past history and urinary tract infection

Characteristics	Bacteriuria			OR (IC:95%)	P
	Positive	Negative	Total		
Sexuality					
< 3times/week	32(10)	287(90)	319(90.1)		
≥ 3times/week	3(8.6)	32(91.4)	35(9.9)	0.834 (0.241–2.882)	0.77
Past history of UTI					
No	21(8)	243(92)	264(74.6)		
Yes	14(15.6)	76(84.4)	90(25.4)	2.183 (1.055–4.518)	**0.04**
Past history of miscarriage					
No	28(10.6)	236(89.4)	264(74.6)		
Yes	7(7.8)	83(92.2)	90(25.4)	1.09 (0.63–1.88)	0.75
Past history of Premature labor					
No	34(9.9)	307(90.1)	341(96.3)		
Yes	1(7.7)	12(92.3)	13(3.7)	0.81 (0.101–6.491)	0.84
HIV^a^					
No	34(10.1)	301(89.9)	335(94.6)		
Yes	1(5.3)	18(94.7)	19(5.4)	0.517 (0.067–4.021)	0.52
BMI^b^					
Normal	13(11.7)	98(88.3)	111(31.4)	1	
Overweigh	11(8.6)	117(91.4)	128(36.2)	0.709 (0.304–1.653)	0.42
Stage 1obesity	6(7.1)	78(92.9)	84(23.7)	0.58 (0.211–1.596)	0.29
Stage 2 obesity	5(18.5)	22(81.5)	27(7.6)	1.713 (0.553–5.306)	0.35
Stage 3 obesity	0(0)	4(100)	4(1.1)	0.03 (0.006–0.1)	0.97

a: Human Immunodeficiency Virus; b: Body Mass Index

The significance of entries in boldface: significant *p* value

## Discussion

In this study, we report a prevalence of 9.9% of bacteriuria among the 354 pregnant women of the 3 hospitals in Douala. The statistically significant predictor of bacteriuria was past history of UTI. High level of education was protective. *E. coli* was the most commonly isolated pathogen.

The prevalence of bacteriuria in pregnant women of the 3 hospitals in Douala was 9.9%. This prevalence is lower than that reported by Mokube et al. in Cameroon (23.5%) [10]. This may be due to the difference in the study population. Women with university level represent half of our study population and the quarter of theirs. Furthermore the study of Mukube et al. was carried out in a rural area while ours was conducted in an urban area. Some authors revealed that women who resided in rural areas were more likely to have ABU when compared to urban dwellers [13]. This may be because of poor hygienic conditions and a lack of social amenities in rural areas. Our prevalence is also lower than that obtained by some other authors [8, 10, 12, 13, 17]. In addition, we had a 5.7% prevalence of asymptomatic bacteriuria in our study population. Our value falls in the range of 2–10% reported elsewhere [6, 7, 10, 18]. Conversely, it is lower than that reported by other authors [8, 13, 14, 19]. Variation in studies may be due to differences in geographical location, socioeconomic status, setting of study (primary care, general hospital and community), sample size and variation in screening tests (cut-off point for the detection of pathogens).

The prevalence of cystitis was higher (3.6%) than that reported in the literature 1–2% [2]. It is not easy to clearly defined signs of cystitis in pregnancy especially during the second and third term when pollakiuria and lower abdominal pains are frequent. Acute pyelonephritis was found in 0.6% which is within the range reported in other studies [5]. Acute pyelonephritis is a severe form of UTI and can lead to maternal (anemia, renal failure and preeclampsia) or fetal (premature delivery) complications. Treating ABU reduces its frequency.

The age, marital status, HIV status, obesity, gestational age, parity and low monthly income did not have any statistical significant influence on bacteriuria in our study. However, level of immunosuppression in people living with HIV/AIDS has been found as a predictor of bacteriuria in pregnancy [16].

High educational level was protective. Educational level attained may be an indicator of the socioeconomic status of the women. Lower levels of education and low socioeconomic status have been related to higher prevalence of ABU in others studies [5, 20]. Another predictor of bacteriuria in pregnancy identified in this study was past history of UTI. Other studies have report a similar result [10, 15, 21, 22]. It is known that some patients are more predisposed to urinary tract infection than others (genetic propensity and anatomical predisposition) [23].

*E. coli* was the most predominant pathogen with an overall isolation rate of 48.6%. Comparable findings have been reported in Ethiopia (47.5%), Khartoum (42.4%), Iran (80%), Cameroon (33%), Nigeria (48%), Pakistan (70%), and India (60.1%) [7, 9–11, 16, 21, 22, 24, 25]. *Klebsiella pneumoniae* was the second most prevalent pathogen in this study with 14.3%. This finding was similar to that reported by other studies where *Klebsiella pneumoniae* was the second or third commonest pathogen [11, 16, 17]. Some authors reported *Staphylococcus aureus* to be the most common pathogen [13, 19]; it accounts for the fourth cause of bacteriuria in our study. Variation in geographical location and uses of antibiotic can account for these differences.

Limits of study: This study is weakened by its hospital-based design, which may not be a true reflection of what is happening in the community. The antibiotic susceptibility of the bacteria isolated was not part of this study but we know that it is important for appropriate treatment of these women.

## Conclusion

Bacteriuria was frequent in pregnant women but the prevalence was low when compared to some African studies. Asymptomatic bacteriuria is more common. Past history of UTI significantly influence the risk of bacteriuria in this study population. High level of education was protective. *E. coli* was the dominant uropathogen isolated among the pregnant women. Education and proper treatment of urinary tract infections should be provided to help reduce the occurrence of bacteriuria in order to prevent its severe complications. A community-based study in this subject matter is also recommended.

## Abbreviations

ABU: Asymptomatic Bacteriuria
AIDS: Acquired Immuno Deficiency Syndrome
CFU: Colony Forming Units
CI: Confidence Interval
CLED: Cystine Lysine Electrolyte Deficient
CSPRO: Census and Survey Processing System
E Coli: Escherichia Coli
EMB: Eosine Methylen Blue
HIV: Human Immunodeficiency Virus
OR: Odd Ratio
SPSS: Statistical Package for the Social Sciences
UTI: Urinary Tract Infection

## References

[1] Fournié A, TJalle T, Sentilhes L. Infections urinaires chez la femme enceinte. EMC-Gynécologie-Obstétrique. 2008:5–047-A10. https://www.em-consulte.com/article/178866/infections-urinaires-chez-la-femme-enceinte.

[2] Société de Pathologie Infectieuse de Langue Française (2015). Recommandations de bonne pratique: Infections urinaires au cours de la grossesse.

[3] Smaill FM, Vazquez JC (2015). Antibiotics for asymptomatic bacteriuria in pregnancy. Cochrane Database Syst Rev.

[4] Smaill F (2007). Asymptomatic bacteriuria in pregnancy. Best Pract Res Clin Obstet Gynaecol.

[5] Wing DA, Fasset MJ, Getahun D (2014). Acute pyelonephritis in pregnancy: an 18-year retrospective analysis. Am J Obstet Gynecol.

[6] Easter SR, Cantonwine DE, Zera CA, Lim KH, Parry SI, McElrath TF (2016). Urinary tract infection during pregnancy, angiogenic factor profiles, and risk of preeclampsia. Am J Obstet Gynecol.

[7] Alemu A, Moges F, Shiferaw Y (2012). Bacterial profile and drug susceptibility pattern of urinary tract infection in pregnant women at university of Gondar teaching hospital, Northwest Ethiopia. BMC Res Notes.

[8] Assefa A, Asrat D, Woldeamanuel Y, G/Hiwot Y, Abdelle A, Melesse T (2008). Bacterial profile and drug susceptibility pattern of urinary tract infection in pregnant women at Tikur Anbessa specialized hospital Addis Ababa, Ethiopia. Ethiop Med J.

[9] Hamdan HZ, Ziad AH, Ali SK, Adam I (2011). Epidemiology of urinary tract infection and antibiotics sensitivity among pregnant women at Kharthoum north hospital. Ann Clin Microbiol Antimicrob.

[10] Marzieh J, Mohsen S, Nasrin R, Koroosh K (2014). Prevalence of urinary tract infection and somes factors affected in pregnant women in Iran, Karaj. Middle-East J Sci Res.

[11] Mokube MN, Atashili J, Halle-Ekane GE, Ikoney MG, Ndumbe MP (2013). Bacteriura amongst pregnant women in the BUEA health district Cameroon: prevalence, predictors, antibiotics susceptibility patterns and diagnosis. PLoS One.

[12] AL-Haddad AM (2005). Urinary tract infection among pregnant women in Al-Mukalla district, Yemen. East Mediterr Health J.

[13] Onu FA, Ajah LO, Ezeonu PO, Umeora OU, Ibekwe PC, Ajah MI (2015). Profile and microbiological isolates of asymptomatic bacteriuria among pregnant women in Abakaliki, Nigeria. Infect Drug Resist.

[14] Colgan R, Nicolle LE, McGlone A, Hooton TM (2006). Asymptomatic bacteriuria in adults. Am Fam Physician.

[15] Emiru T, Beyene G, Tsegaye W, Melaku S (2013). Associated risk factors of urinary tract infection among pregnant women at Felege Hiwot referral hospital, Bahir Dar, north West Ethiopia. BMC Res Notes.

[16] Awolude OA, Adesina OA, Oladokun A, Mutiu WB, Adewole IF (2010). Asymptomatic bacteriuria among HIV positive pregnant women. Virulence.

[17] Okonko IO, Ijandipe LA, Ilusanya OA (2009). Incidence of urinary tract infection among pregnant women in Ibadan, South-Western Nigeria. Afr J Biotechnol.

[18] Labi AK, Yawson AE, Ganyaglo GY, Newman MJ (2015). Prevalence and associated risk factors of asymptomatic bacteriuria in antenatal clients in a large teaching hospital in Ghana. Ghana Med J.

[19] Tadesse E, Teshome M, Merid Y, Kibret B, Shimelis T (2014). Asymptomatic urinary tract infection among pregnant women attending the antenatal clinic of Hawassa referral hospital, southern Ethiopia. BMC Res Notes.

[20] Ojide CK, Wagbatsoma VA, Kalu EI, Nwadike VU (2014). Asymptomatic bacteriuria among antenatal care women in a tertiary hospital in Benin, Nigeria. Niger J Exp Clin Biosci.

[21] Haider G, Zehra N, Munir AA, Haider A (2010). Risk factors of urinary tract infection in pregnancy. J Pak Med Assoc.

[22] Rajaratnam A, Baby NM, Kuruvilla TS, Machado S (2014). Diagnosis of asymptomatic bacteriuria and associated risk factors among pregnant women in Mangalore, Karnataka, India. J Clin Diagn Res.

[23] Minardi D, D’Anzeo G, Cantoro D, Conti A, Muzzonigro G (2011). Urinary tract infections in women: etiology and treatment options. Int J Gen Med.

[24] Oladeinde BH, Omoregie R, Oladeinde OB (2015). Asymptomatic urinary tract infection among pregnant women receiving antenatal care in a traditional birth home in Benin city, Nigeria. Ethiop J Health Sci.

[25] Thakre SS, Dhakne SN, Thakre SB, Ughade SN (2015). Hygiene practices and sexual activity associated with urinary tract infection in rural pregnant women of Nagpur, India. Indian J Med Microbiol.

